# P13 Initiating pre-travel vaccination: a critical step for transitioning from the yellow fever vaccination centre to the travel clinic: record-based analysis at a tertiary care institute, Northern India

**DOI:** 10.1093/jacamr/dlag102.019

**Published:** 2026-06-26

**Authors:** Smita Sinha, Ashwani Seth, Vartika Saxena

**Affiliations:** Department of Community Medicine, All India Institute of Medical Sciences Rishikesh India; Department of Community Medicine, All India Institute of Medical Sciences Rishikesh India; Department of Community Medicine, All India Institute of Medical Sciences Rishikesh India

## Abstract

**Background:**

As of 2023, around 22 million Indian nationals are travelling to foreign countries. According to the Travel and Development Tourism Index 2024, India is ranked 39^th^ among 119 countries. Given the mandatory yellow fever vaccination requirement in certain countries, the vaccine is acceptable. A significant proportion of these travellers often return with other illnesses like traveler’s diarrhoea, febrile illness, Hepatitis A and B infection, and typhoid. This warrants the need for transitioning the yellow fever vaccination centre to a travel clinic. To start with, the pre-travel vaccination is one of the important strategies to address this issue.

**Objectives:**

To estimate the proportion of travellers accepting the pre-travel vaccination and to identify the reasons for non-acceptance of the pre-travel vaccination.

**Material and methods:**

The process of transitioning towards the travel clinic was initiated in December 2026. A secondary data analysis was performed on the data from 1^st^ January to 31^st^ January 2026. The data is maintained by the designated nursing officer of the adult vaccination centre under the Department of Community Medicine, AIIMS Rishikesh. The data extracted from record were age, sex, address, occupation, country of travel, vaccine advised, vaccine administered, and reason for not getting vaccinated. The data was obtained in MS Excel and analysed using R Studio. The acceptance of the pre-travel vaccination and the reasons for non-acceptance were presented as frequencies and proportions. The pre-travel vaccination was defined as the administration of country specific vaccine other than the mandatory vaccines for travel, like the yellow fever vaccine and oral polio vaccine. The pre-travel vaccination was categorized as complete (if all advised vaccines were administered) and partial (if any of the advised vaccines were not administered). The pre-travel vaccination was advised as per the Centre for Disease Control and Prevention (CDC) guidelines 2025.

**Results:**

Around 253 individuals were vaccinated in the adult vaccination clinic, of which 208 (82.2%) individuals were travelling to foreign countries. The mean (SD) age of the individuals was 31.7 (4.2) years. Of the total individuals, 213 (84.2%) were male. Of those travelling to foreign countries, around 34 (16.3%) individuals were travelling to South Africa, 26 (12.2%) to the US, 24 (11.3%) to Kenya, and the rest to other countries like Congo, Ghana, Nigeria, and Tanzania in the majority. The individuals eligible requiring pre-travel vaccination were 158 (76.0%), and among them, the complete acceptance of pre-travel vaccination was by 133 (84.2%), and partial acceptance by 10 (6.3%). All (100%) of the individuals received the yellow fever vaccine. The acceptance (received/advised) for Td was 93% (40/43), for MMR 93% (27/29), for Hepatitis B 92% (46/50), Influenza 91% (10/11), Typhoid vaccine 90% (19/21), and others were even lower. The reason for non-acceptance was refusal by the patients (3.8%) and unaffordability (5.7%).

**Conclusions:**

Approximately two-thirds of the individuals were eligible for pre-travel vaccination, of which the majority either completely or partially accepted vaccination. Awareness of pre-travel vaccination and lower cost of the vaccines would improve the acceptance. Hence, adopting the pre-travel vaccination strategy would help us transition from the yellow fever centre to a travel clinic.

